# CTCA for detection of significant coronary artery disease in routine TAVI work-up: A systematic review and meta-analysis

**DOI:** 10.1007/s12471-018-1149-6

**Published:** 2018-09-03

**Authors:** T. P. W. van den Boogert, J. Vendrik, B. E. P. M. Claessen, J. Baan, M. A. Beijk, J. Limpens, S. A. M. Boekholdt, R. Hoek, R. N. Planken, J. P. Henriques

**Affiliations:** 1 Heart Centre, Academic Medical Centre, part of the Amsterdam Cardiovascular Sciences, University of Amsterdam, Amsterdam, The Netherlands; 2 Medical Library, Academic Medical Centre, University of Amsterdam, Amsterdam, The Netherlands; 3 Department of Radiology and Nuclear Medicine, Academic Medical Centre, University of Amsterdam, Amsterdam, The Netherlands

**Keywords:** Coronary artery disease, Aortic stenosis, Computed tomography coronary angiography, Diagnostic accuracy, Coronary angiography, Transcatheter aortic valve implantation

## Abstract

Transcatheter aortic valve implantation (TAVI) has evolved to standard treatment of severe aortic stenosis in patients with an intermediate to high surgical risk. Computed tomography coronary angiography (CTCA) could partially replace invasive coronary angiography to diagnose significant coronary artery disease in the work-up for TAVI. A literature search was performed in MEDLINE and EMBASE for papers comparing CTCA and coronary angiography in TAVI candidates. The primary endpoint was the diagnostic accuracy of CTCA, compared to coronary angiography, for detection of significant (>50% diameter stenosis) coronary artery disease, measured as sensitivity, specificity, positive—(PPV) and negative predictive value (NPV). Seven studies were included, with a cumulative sample size of 1,275 patients. The patient-based pooled sensitivity, specificity, PPV and NPV were 95, 65, 71 and 94% respectively. Quality assessment revealed excellent and good quality in terms of applicability and risk of bias respectively, with the main concern being patient selection. In conclusion, on the basis of a significance cut-off value of 50% diameter stenosis, CTCA provides acceptable diagnostic accuracy for the exclusion of coronary artery disease in patients referred for TAVI. Using the routinely performed preoperative computed tomography scans as a gatekeeper for coronary angiography could decrease additional coronary angiographies by 37% in this high-risk and fragile population.

**Electronic supplementary material:**

The online version of this article (10.1007/s12471-018-1149-6) contains supplementary material, which is available to authorized users.

## Introduction

Severe aortic valve stenosis is found in 3.4% of patients over 75 years old [1–3]. Transcatheter aortic valve implantation (TAVI) has evolved to standard treatment of aortic valve stenosis in patients with an intermediate to high surgical risk [2, 3]. Pre-procedural screening for coronary artery disease (CAD) is recommended in current guidelines, due to its high prevalence (40 to 75%) and possible harmful influence on procedural outcome and prognosis if left untreated [4]. Computed tomography (CT) is part of the routine preoperative work-up for assessment of the access route and for sizing the valve prosthesis. The available CT images, however, also allow for assessment of the coronary arterial tree.

In a previous systematic review of patients undergoing conventional surgery for valvular disease, Opolski et al. found a sensitivity of 94% to rule out CAD using computed tomography coronary angiography (CTCA) when using ≥64 detector row CT scanners [5]. A potentially important limitation of CTCA applied in the TAVI population is the anticipated high coronary artery calcium load that may result in lower diagnostic accuracy due to blooming artefacts and beam hardening [6]. Furthermore, due to the possible clinical harm, aortic valve stenosis patients do not receive per protocol nitroglycerin prior to the CT scan, which further impedes diagnostic evaluation of the coronary arteries [7]. On the contrary, patients undergoing TAVI are almost exclusively elderly, fragile patients and would strongly profit from such a single non-invasive diagnostic approach.

The objective of this systematic review was to summarise the available diagnostic accuracy for CTCA to detect significant (>50% stenosis) CAD in patients referred for TAVI and to investigate the possibility to safely use CTCA as a gatekeeper for coronary angiography in the TAVI work up.

## Methods

### Literature search and study selection

This systematic review was conducted and reported according to the protocol specified in the Preferred Reporting Items for Systematic Reviews and Meta-analyses statement [8]. A clinical librarian (JL) performed a systematic search in OVID MEDLINE (including Epub Ahead of Print, In-Process & Other Non-Indexed Citations) and OVID EMBASE of studies published between January 1, 1946 to December 23, 2017 to find studies evaluating the diagnostic accuracy of CTCA vs. coronary angiography for the evaluation of CAD in patients receiving TAVI. We searched for the concepts TAVI and CTCA, using controlled terms like MesH and text words. No language, date or other restrictions were applied. Reference lists and the citing articles of the identified relevant papers were cross-checked in Web of Science. The bibliographic records we retrieved were imported and de-duplicated in ENDNOTE (Clarivate analytics 2017, Philadelphia PA, USA). The complete search strategies are presented in appendices in the supplementary material as supplementary Tab. 1 and 2. Three investigators (TvdB, JV, RH) independently screened all titles and abstracts. Potentially eligible studies were retrieved and reviewed in full text. Papers were excluded if they were not reporting original data of patients who received both pre-procedural multi-detector CT (≥64 detector rows) and coronary angiography for the evaluation of CAD in the work-up of TAVI. Discrepancies regarding inclusion or exclusion of a study were resolved by consensus.

### Data extraction and data analysis

The primary endpoint of this systematic review was the diagnostic accuracy of pre-procedural CTCA, compared with pre-procedural coronary angiography, for the evaluation of CAD in patients receiving TAVI. Diagnostic accuracy was defined as the sensitivity, specificity, positive—(PPV) and negative predictive value (NPV). Three investigators (TvdB, JV, RH) independently performed data extraction from the selected studies using a standardised form for data extraction. Differences between reviewers were resolved by consensus. The methodological quality of included studies was assessed using the modified Quality Assessment of Studies of Diagnostic Accuracy Included in Systematic Reviews-2 criteria (QUADAS-2) by 2 independent reviewers (TvdB, JV). The meta-analysis of the primary endpoint was performed on a per patient level. Sensitivity, specificity, PPV and NPV were extracted or computed based on true-positive, true-negative (TN), false-positive (FP), and false negative (FN) rates for all studies independently and combined. Clinical heterogeneity was assessed by a qualitative comparison of the methods and baseline characteristics of the study population in the individual studies. Statistical heterogeneity was assessed using the bivariate model [9]. Subgroups were analysed for their influence on diagnostic accuracy outcome by comparing summary receiver operator characteristics (SROC) curves. Two subgroups were stratified, based on rotation time of the CT scanner and prevalence of CAD. Data analysis was performed using the statistical software R version 1.0.136 (R Foundation for Statistical Computing, Vienna, Austria), employing the Meta-Analysis of Diagnostic Accuracy ‘mada’ package.

## Results

### Study selection

**Fig. 1:**
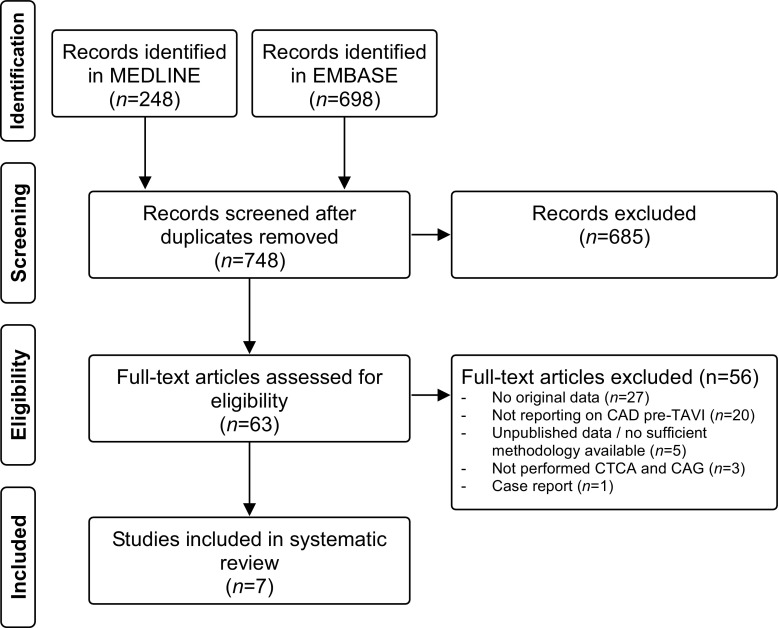
Flowchart of selection process. Scheme, depicting study identification and selection process. (*CAD* coronary artery disease, *CAG* coronary angiography, *CTCA* computed tomography coronary angiography, *TAVI* transcatheter aortic valve implantation)

Of the 946 references identified by the electronic search (Fig. 1), 63 articles were potentially eligible. A total of 7 papers were included in the final analysis. We excluded 56 references due to the following reasons: i) the paper did not analyse original data (*n* = 27); ii) the paper did not report on CAD in the work-up of TAVI patients but specifically on valve selection and valve sizing (*n* = 20); iii) unpublished data without complete methodology (*n* = 5); iv) not routinely performed CTCA and coronary angiography (*n* = 3); v) the paper reported on a single case (*n* = 1).

### Study characteristics

Baseline and CT scan characteristics are listed in Tab. 1, 2 and supplementary Tab. 3. The combined studies included 1,275 patients with a mean age of 81.5 years and 42.7% of patients were male. Six studies reported BMI, with a mean BMI of 26.5 kg/m^2^. In the studies (*n* = 6) reporting co-morbidities, 28.3% of the population had diabetes mellitus and 24.7% had atrial fibrillation. Known CAD was present in 25.7% of the population, for which 27.0% underwent previous percutaneous coronary intervention (PCI) and 16.4% previous coronary artery bypass grafting (CABG). All studies used a retrospective electrocardiogram-gated protocol. Six studies reported on CT settings. The scans were acquired at 100 to 120 kilovolts (kV) and 185–600 mA per rotation. The amount of contrast used varied between 60 and 120 ml. The iodine concentration of the contrast medium used varied between 300 and 400 mg I/ml. Mean heart rate was reported in 5 studies and varied between 61 beats/min and 74 beats/min. All studies used a cut-off value of >50% diameter stenosis to determine the presence of significant CAD.

**Table 1:** Baseline characteristics

	*N*	Age (years)	Men (%)	BMI (kg/m^2^)	DM (%)	AF (%)	HC (%)	HT (%)	Smoking (%)	CAD (%)	PCI (%)	CABG (%)
Pontone et al. (2011) [13]	60	80	36.6	25.0	13.3	NR	40.0	66.7	25.0	36.7	23.3	16.7
Andreini et al. (2014) [18]	325	81.1	40.6	25.6	30.2	NR	53.8	74.8	20.0	NR	15.0	12.9
Hamdan (2015) [19]	115	80.4	43.4	26.8	30.4	7.8	70.4	85.2	36.5	52.1	29.5	20.0
Opolski (2015) [20]	475	82	41.0	27.5	31.6	18.9	48.2	94.7	NR	NR	47.6	19.2
Harris et al. (2015) [21]	100	79.6	61.0	NR	24.0	36.0	72.0	92.0	59.0	NR	16.0	41.0
Matsumoto (2017) [10]	60	84.4	28.3	22.2	NR	NR	NR	NR	NR	24.0	10.0	3.3
Rossi et al. (2017) [22]	140	82.3	48.6	27.1	20.7	31.4	59.3	75.0	19.3	0	0	0
*Mean of total*	*182.1*	*81.5*	*42.7*	*26.5*	*28.3*	*24.7*	*54.6*	*84.6*	*28.1*	*25.7*	*27.0*	*16.4*

Baseline characteristics are given per individual study and as a mean of the total of the studies combined

*AF* atrial fibrillation, *BMI* body mass index, *CABG* coronary artery bypass grafting, *CAD* coronary artery disease, *DM* diabetes mellitus, *HC* hypercholesterolaemia/hyperlipidaemia, *HT* hypertension, *N* number of studied subjects, *NR* not reported, *PCI* percutaneous coronary intervention

**Table 2:** CT scan characteristics

	Detector rows (slices)	Rot. Time (ms/rot)	Tube voltage (kV)	Tube charge (mAs)	Contrast conc. (mg I/ml)	Contrast volume (ml)	Mean HR (/min)	Mean DLP (mGy*cm)	Nitroglycerine	HR control
Pontone et al. (2011) [13]	64 (64)	350	120	650	400	130	NR	NR	NR	Yes
Andreini et al. (2014) [18]	64 (64)	350	100–120	500–600	400	130	61	1,136 ± 275	NR	Yes
Hamdan (2015) [19]	128 (256)	330	100	485	350	65–80	70.4	1,228 ± 386	No	Yes
Opolski (2015) [20]	2 × 40 (2 × 64)	330	120	320–400	NR	80–120	74	2,336 ± 1,036	No	No
Harris et al. (2015) [21]	2 × 64 (2 × 128)	285	NR	NR	320	60	NR	1,279 ± 521	NR	No
Matsumoto (2017) [10]	320 (640)	275	100	185–580	350/370	^a^	70.9	1,281 ± 196	No	No
Rossi et al. 2017 [22]	2 × 64 (2 × 128)	285	100–120	320–400	300	80	70.0	NR	No	No

All studies reported a retrospective ECG-gated scan protocol

*DLP* dose length product, *HR* heart rate, *kV* kilovolt, *mAs* milliampere per rotation, *mg* *I/ml* milligrams of iodide per millilitre, mGy*cm milligray per centimetre, *ml* millilitre

^a^Matsumoto described an algorithm for contrast volume administration: scan time × patient weight × 0.06

### Risk of bias within studies

**Fig. 2:**
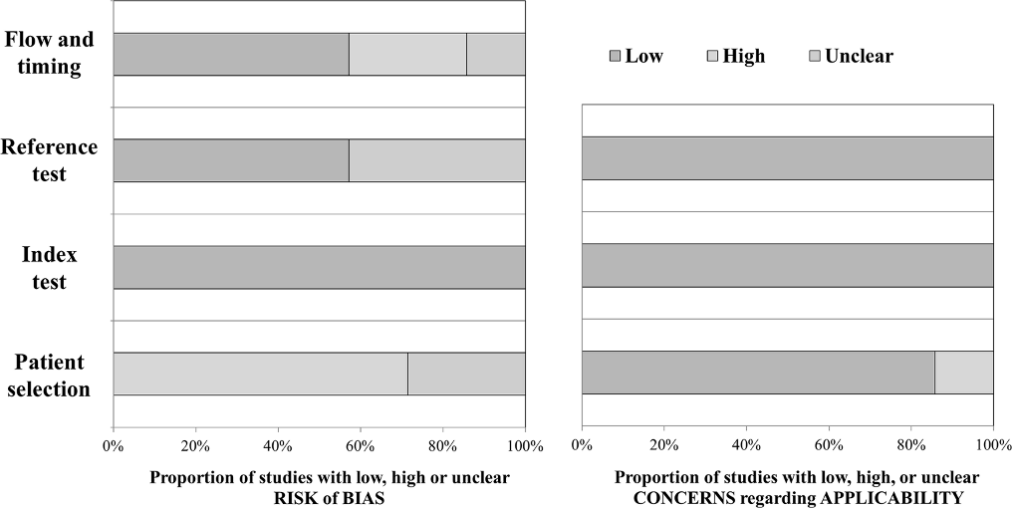
Methodological quality assessment of included studies by QUADAS II. Summary of quality assessment. Low, high or unclear risk of bias or concerns regarding applicability is represented by green, red or blue respectively. (*QUADAS-2* Quality Assessment of Studies of Diagnostic Accuracy Included in Systematic Reviews 2)

Overall, the selected studies showed excellent quality in terms of applicability. Risk of bias within the studies was scored as acceptable quality, with the main concern being patient selection (Fig. 2). Quality assessment of individual studies is shown in supplementary Tab. 4 and elaborated in the supplementary text (Risk of bias within studies). For more insight into patient selection, all inclusion and exclusion criteria of the individual studies and the studies combined are listed in supplementary Tab. 5 and summarised in supplementary Fig. 1.

### Results of individual studies

**Fig. 3:**
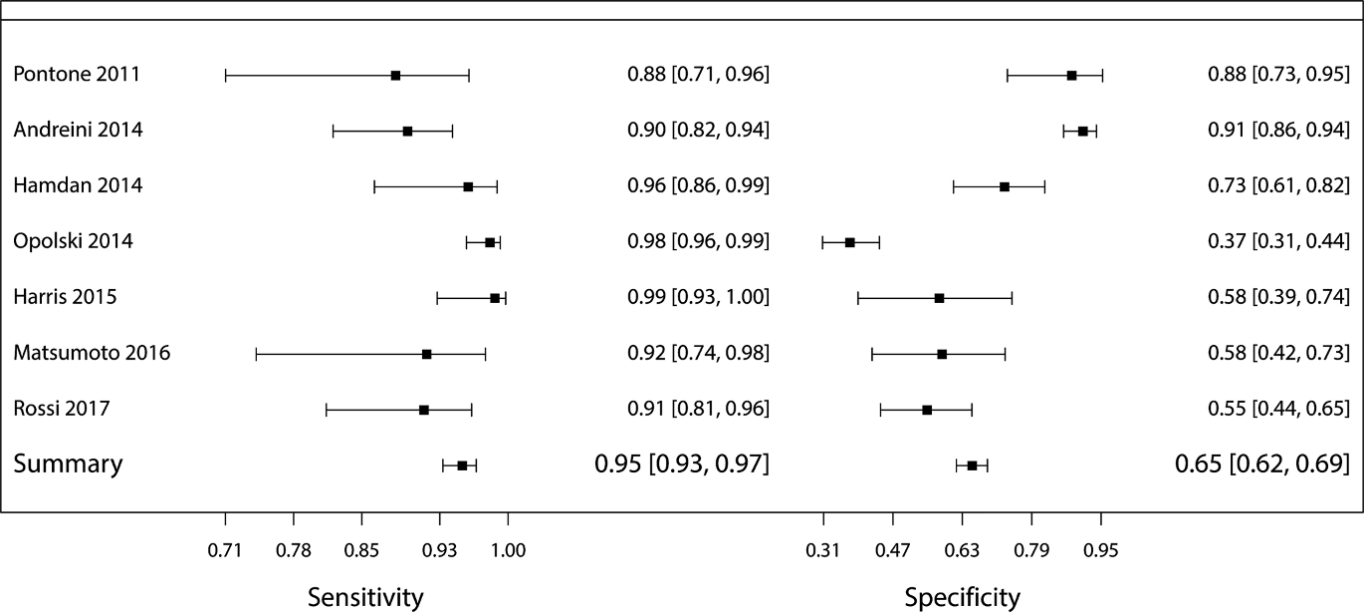
Diagnostic accuracy paired forest plot. Sensitivity and specificity of CTCA versus CAG for the detection of CAD in patients receiving TAVI. Results are depicted in a paired forest plot, with resulting confidence intervals for each individual study and for the studies combined

The results of the individual papers are listed in Tab. 3. Study sample size varied between 60 and 475 patients. The prevalence of CAD varied between 29.8 and 74.0%. The percentages of true positive and true negative varied between 26.8 and 73.0% and between 15.0 and 63.7% respectively. The percentage of false positives and false negatives varied between 6.5 and 27.2% and between 1.0 and 5.0% respectively. The resulting sensitivity and specificity varied between 88.5 and 98.5% and between 37.1 and 90.8% respectively. The PPV and NPV varied between 58.9 and 86.9% and between 90.0 and 96.0% respectively. Fig. 3 shows a paired forest plot of the sensitivity and specificity with resulting confidence intervals of the individual studies and the studies combined.

**Table 3:** Diagnostic value of CTCA

	*N*	Prev (%)	TP (%)	TN (%)	FP (%)	FN (%)	Sensitivity	Specificity	PPV	NPV
Pontone et al. (2011) [13]	60	26 43.3%	23 38.3%	30 50.0%	4 6.7%	3 5.0%	88.5%	88.2%	85.2%	90.9%
Andreini et al. (2014) [18]	325	97 29.8%	87 26.8%	207 63.7%	21 6.5%	10 3.1%	89.7%	90.8%	80.6%	95.4%
Hamdan (2015) [19]	115	49 42.6%	47 40.9%	48 41.7%	18 15.7%	2 1.7%	95.9%	72.7%	72.3%	96.0%
Opolski (2015) [20]	475	270 56.8%	265 55.8%	76 16.0%	129 27.2%	5 1.1%	98.1%	37.1%	67.3%	93.8%
Harris et al. (2015) [21]	100	74 74.0%	73 73.0%	15 15.0%	11 11.0%	1 1.0%	98.6%	57.7%	86.9%	93.8%
Matsumoto (2017) [10]	60	24 40.0%	22 36.7%	21 35.0%	15 25.0%	2 3.3%	91.7%	58.3%	59.5%	91.3%
Rossi et al. (2017) [22]	140	58 41.4%	53 37.9%	45 32.1%	37 26.4%	5 3.6%	91.4%	54.9%	58.9%	90.0%
*Total*	*1,275*	*598* *46.9%*	*570* *44.7%*	*442* *34.7%*	*235* *18.4%*	*28* *2.2%*	*95.3%*	*65.3%*	*70.8%*	*94.0%*

Outcomes of individual studies and of the studies combined are listed as integers and as a percentage

*FN* false negatives, *FP* false positives, *N* number of studied subjects, *NPV* negative predictive value, *PPV* positive predictive value, *Prev* prevalence of coronary artery disease as reported, *TN* true negatives, *TP* true positives

### Synthesis of results

The total amount of true-positive, true-negative, false-positive and false-negative findings was 570, 442, 235 and 28 respectively. The resulting accuracy measures comprising the primary endpoint, i. e. sensitivity, specificity, PPV and NPV were 95.3% (95% confidence interval [CI] 93.3 to 96.9%), 65.3% (95% CI 61.6 to 68.9%), 70.8% (95% CI 68.6 to 72.9%) and 94.0% (95% CI 91.6 to 95.8%) respectively.

In 1,012 patients (79.4%), there was agreement between CTCA and coronary angiography on the presence of significant (>50% stenosis) CAD. Of the 263 patients with disagreement between CTCA and coronary angiography, the vast majority (*n* = 235, 89%) had false-positive CTCA findings as they tested negative on coronary angiography. Most important, only 28 patients (2.8%) had false-negative findings and tested positive on coronary angiography.

### Heterogeneity assessment

**Fig. 4:**
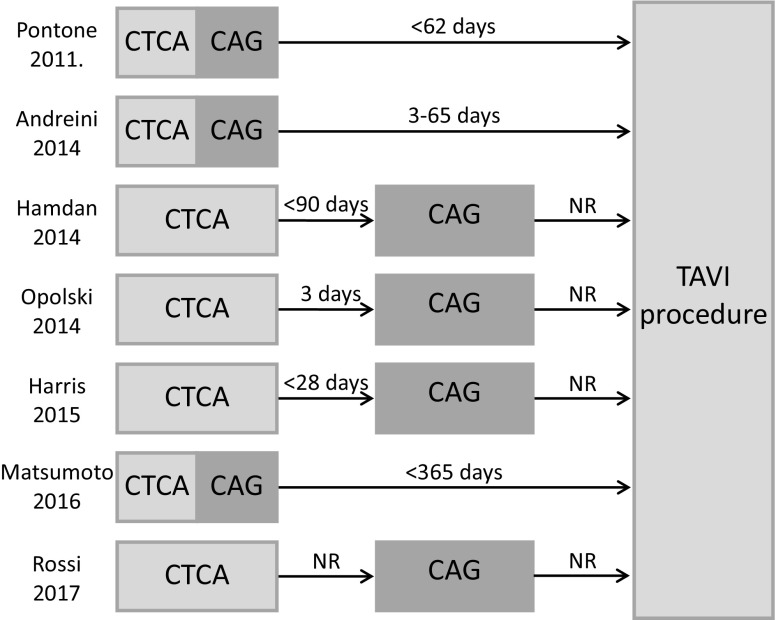
Flow and timing. Scheme, depicting the timing of the pre-procedural CTCA and CAG before TAVI. (*CTCA* computed tomography coronary angiography, *CAG* coronary angiography, *NR* not reported, *TAVI* transcatheter aortic valve implantation procedure)

Regarding baseline characteristics, the included studies were clinically homogenous regarding age, BMI and comorbidities with random variation consistent with a normal TAVI population. All studies used a reduction of 50% diameter stenosis as a threshold for significant CAD. The percentage of known CAD varied between studies (0–52.1%) and is clinically relevant as it will alter the pre-test probability. The tested percentage of significant (>50% stenosis) CAD during study varied between (29.8–74%) and was interpreted as clinically relevant. The methods were heterogeneous regarding the time between CT and coronary angiography (3–365 days) (Fig. 4), contrast administration (60–120 ml with a varying iodine concentration between 300 and 400 mg I/ml and different contrast administration protocols) and scanner specifications and settings (Tab. 2 and supplementary Tab. 3).

**Fig. 5:**
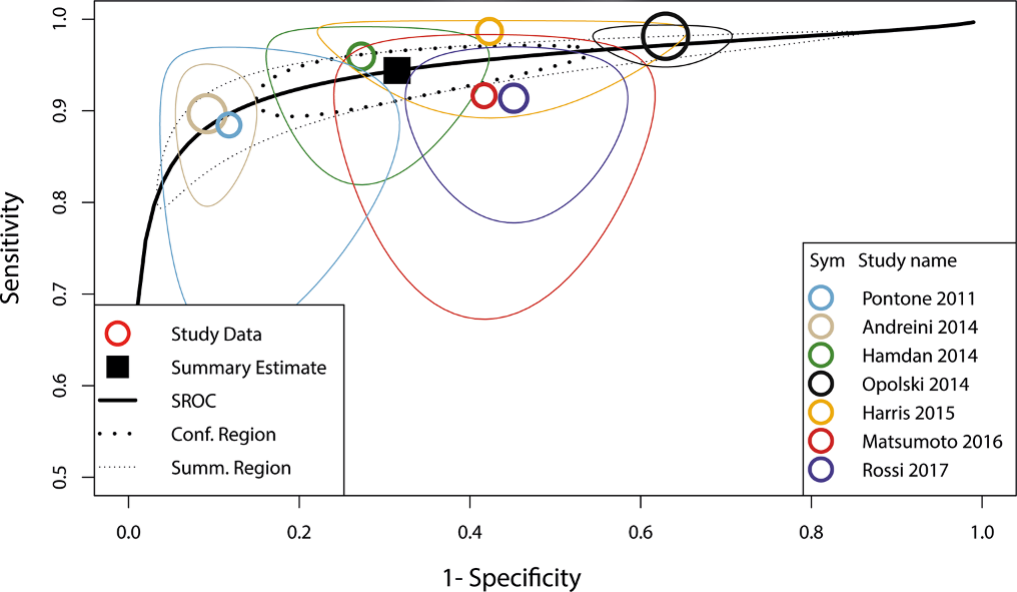
Summary receiver operator curve plot, bivariate model. Sensitivity versus false positive rate is plotted in a for all included studies. Each study is represented by a coloured circle, size being dependent on study size. The black square represents the summary estimate. The thick dashed lines represents the 95% confidence region (Conf. Region) and the thin dashed line represents the 95% summary region (Summ. Region). (*SROC* summary receiver operator characteristic curve, *Sym* symbol)

Statistical heterogeneity assessment, using the bivariate model is shown in Fig. 5. The sensitivity was plotted against the 1‑specificity (false-positive rate) of the included studies. The summary estimate of the included studies is shown with resulting confidence and summary region. The prediction region predicts a 95% confidence region for the true sensitivity and specificity of a future study. All included studies were enclosed in, or visually close to this prediction region and SROC curve and all confidence intervals of the included studies overlapped the SROC curve and the prediction region of the summary estimate. This means that there is low suspicion of statistical heterogeneity and that all studies observed statistically similar results for the diagnostic accuracy measures of CTCA.

### Subgroup analysis

Subgroup analysis is shown in the supplementary material. Here, the SROC curves of different subgroups can be appreciated. The diagnostic accuracy was not significantly different in the subgroups of >300 ms and <300 ms rotation time (Supplementary Fig. 2). The diagnostic accuracy was significantly different in the subgroups of <50% and ≥50% prevalence of CAD (Supplementary Fig. 3). The confidence intervals of both summary estimates did not overlap.

## Discussion

This systematic review and meta-analysis summarised the literature on the diagnostic accuracy for CTCA to detect CAD in patients referred for TAVI on scanners with ≥64 detector rows. The results show that CTCA provides clinical acceptable diagnostic accuracy for the exclusion of significant CAD, due to a high sensitivity and negative predictive value. This meta-analysis of the available data of the 7 included studies (*n* = 1,275) resulted in a sensitivity, specificity, PPV and NPV of 95.3% (95% CI 93.3% to 96.9%), 65.3% (95% CI 61.6 to 68.9%), 70.8% (95% CI 68.6 to 72.9%) and 94.0% (95% CI 91.6 to 95.8%) respectively.

Baseline characteristics of the combined study groups were comparable with registries reporting on TAVI patients [11, 12]. The prevalence of CAD in the combined study groups was 46.9% which is in accordance with CAD prevalence of 40 to 75% in a TAVI population [4]. All but one [13] studies reported the time between CTCA and coronary angiography. The maximum time between CTCA and coronary angiography was limited to one year, which is unlikely to result in interval progression of CAD (Fig. 4). The overall quality of the included studies showed excellent quality in terms of applicability. Overall risk of bias was acceptable. The main concern was patient selection as it influenced the prevalence of known CAD and the pre-test probability of CTCA to find CAD. This could have impacted the diagnostic accuracy measures of CTCA, because a lower prevalence of known CAD is associated with a higher amount of true negatives and better NPV for CTCA. The studies showed excellent uniformity in use of the index and reference test, cut-off value, diagnostic accuracy measures and statistical analysis. Heterogeneity assessment, assessed by visual rating of the bivariate model, yielded acceptable results in terms of homogeneity among the included studies.

The outcome of the primary endpoint differed according to the reported prevalence of CAD. The different prevalence of CAD in the population (<50% and >50% prevalence) resulted in a significant alteration of the SROC curve (Supplementary Fig. 3). The population with a higher prevalence of CAD showed an increase in the number of false-positive results. This can be explained by the tendency of CTCA to overestimate the severity of CAD due to blooming artefacts from calcified stenosis and because studies scored unevaluable coronary artery segments as positive (>50% stenosis).

The mean dose length product (DLP) of the reported studies varied between 1,002 and 2,336 mGy/cm. The concentrations and the amount of contrast used are comparable with coronary angiography.

### Clinical implications

Invasive coronary angiography contributes to patient burden and consumes hospital resources in the work-up for TAVI. It increases the risks of complications, is time consuming and is overall more expensive compared with CTCA [14]. The risks of complications increases with age which is of clinical significance in an almost exclusively elderly, fragile population [15]. Since screening for CAD and CT imaging for pre-procedural planning are both recommended before TAVI procedure [2, 3], the combined use of multi-detector CT for the evaluation of CAD and pre-procedural planning seems practical provided an adequate assessment can be made. An additional coronary angiography could be avoided when significant CAD can be ruled out by CTCA. Reducing the number of coronary angiographies would reduce the risk of complications and reduce the amount of contrast used in an elderly population who often have numerous comorbidities and a high-risk profile for invasive procedures and who are susceptible to contrast-induced nephropathy. In the investigated subjects, CTCA was negative in 470 patients (36.9%) of the patients included in the final analysis. Of the patients with negative findings on CTCA, 94.0% were correctly classified as negative (<50% diameter stenosis), with coronary angiography as a reference standard. The relatively low number of FN is acceptable, given that the cut-off value of 50% reflects relatively mild stenosis. The only study reported on clinical consequences of the false-negative CTCA findings reported no clinical implications regarding revascularisation [10].

At present, European guidelines recommend that PCI of coronary artery stenosis of more than 70% in a proximal segment should be considered in patients receiving TAVI (class IIA, level of evidence C) [4]. The 2017 ACC Expert Consensus guideline states that concurrent coronary revascularisation may be needed, particularly if multi-vessel or left main coronary disease is present, although it is unclear if 30-day mortality is influenced by revascularisation status [2]. In a cohort study conducted by Shamekhi et al., the anatomic severity of CAD was associated with lower survival after TAVI, but not significantly improved by revascularisation [16]. In a retrospective analysis of Paradis et al., the severity of CAD and the completeness of revascularization after PCI or CABG were not associated with lower rates of cardiovascular mortality at both 30 days and 1 year [17]. Therefore, the currently available clinical data do not indicate a clear benefit of pre-TAVI coronary revascularisation. Alternatively, patients can undergo PCI in a separate procedure if anginal complaints persist after TAVI.

### Future perspectives

Technical improvements have already resulted in scanners with higher temporal resolution and the use of iterative reconstruction and advanced image-processing algorithms have resulted in fewer artefacts. Furthermore, improvements in CT acquisition protocols resulted in improved image quality, a lower contrast dose and lower radiation dose. These improvements, and the use of standardised patient specific CT acquisition protocols will further improve the diagnostic properties of CTCA in the future. Furthermore, transcatheter valves are evolving and are used in younger patients stratified in lower-risk groups, possibly making the use of nitroglycerin and heart rate control for CTCA more feasible. This will result in better diagnostic capabilities of CTCA before TAVI.

### Limitations

In this systematic review, a total of 4 (out of 7) studies reported on the diagnostic accuracy of CTCA using scanners with >300 ms rotation time [13, 18–20]. When compared with the current generation CT-scanners, these scanners provide lower temporal resolution. This could have affected the overall diagnostic accuracy of CTCA in this systematic review [14]. All the included studies used a different protocol, regarding scanner settings, contrast injection and total amount of contrast used. This impedes the possibility to give any recommendation on protocols for optimal diagnostic accuracy for the detection of CAD in patients receiving TAVI. Furthermore, all 7 included studies had a retrospective design with variable criteria for patient selection, which increased the risk of bias [10, 13, 18–22]. There were some differences in individual studies with respect to the percentage of male patients, prevalence of comorbidities and known CAD. Furthermore, the total amount of included studies is too small for proper subgroup analysis of all covariates. The subgroup analysis performed is an analysis of the most obvious subgroups and is submissive to random variation between the studies.

## Conclusion

On the basis of a cut-off for significance of 50% diameter stenosis, CTCA provides acceptable diagnostic accuracy for the exclusion of significant CAD in patients referred for TAVI. Using the routinely performed preoperative CT scans as a gatekeeper for coronary angiography in the work-up for TAVI could decrease the number of additional coronary angiographies by 37% in this high-risk population.

## Caption Electronic Supplementary Material

Suppl. Table 1 Search (MEDLINE)

Suppl. Table 2 Search (EMBASE)

Suppl. Table 3 CT scan characteristics (Supplement)

Suppl. Table 4 Methodological quality assessment of included studies by QUADAS-2

Suppl. Table 5 Summary of inclusion and exclusion of patients

Supplementary text—Risk of bias within studies

Suppl. Fig. 1 Total inclusion and exclusion of patients

Suppl. Fig. 2 Subgroup analysis for CT-scanner rotation times

Suppl. Fig. 3 Subgroup analysis for CAD prevalence
