# Supplementary material for: CTCA for detection of significant coronary artery disease in routine TAVI work-up: A systematic review and meta-analysis
Source: Neth Heart J. 2018 Sep 3;26(12):591–9. doi: 10.1007/s12471-018-1149-6 (PMC6288031; doi:10.1007/s12471-018-1149-6)
Supplement: Supplementary file 1 — Suppl. Table 1 Search (MEDLINE) [file 12471_2018_1149_MOESM1_ESM.doc]

**Supplementary T**able 1 Search (MEDLINE)

| **#** | **Searches** | **Results** |
| --- | --- | --- |
| 1 | Transcatheter Aortic Valve Replacement/ | 2618 |
| 2 | (TAVI or T-AVI or preTAVI or TA-ViVI or TAViVI or TA-AVI or TF-AVI or TFAVI or TAP-API or TAVR or TAVRs or preTAVR or TA-AVR or TFAVR or TF-AVR or TV-AVI or TVAVI or TAOAVI* or TAO-AVI* or TV-AVR* or TVAVR* or PAVI or P-AVI or PAVR or P-AVR).tw,kf. | 5231 |
| 3 | (TATM or T-AVR or THV).tw,kw. and (aort* or (heart valve adj2 (implant* or replac* or preimplant*))).mp. | 223 |
| 4 | ((percutan* or per-cutan* or transcutan* or trans-cutan* or transcath* or trans-cath* or transapic* or trans-apic* or transfemor* or trans-femor* or transsubclav* or subclav* or transaort* or trans-aort* or transvasc* or transvasc*) adj4 (AVR or AVI or AVRs or AVIs or (aort* adj (valve or valves) adj4 (implant* or preimplant* or replac* or intervent* or insert* or repair*)))).tw,kf. | 7579 |
| **5** | **or/1-4 [TAVI/TAVR]** | **8439** |
| 6 | computed tomography angiography/ | 3676 |
| 7 | (tomography, x-ray computed/ or exp tomography, spiral computed/ or ((computed adj3 tomogra*) or variable helical pitch or (VHP adj3 (imag* or scan*)) or ((dual-source or high pitch or highpitch or multimodalit* or multidetector* or multislic* or (multi* adj3 (slic* or detector*))) adj6 (tomogra* or CT or CTs)) or MDCT* or MSCT*).tw,kf.) and (coronary angiography/ or ((coronar* or non-invasiv* or noninasiv*) adj3 angiogra*).tw,kf.) | 14706 |
| 8 | (((comput* adj2 tomograph*) or CT or CTs or MDCT* or MSCT*) adj3 angio*).tw,kf. | 30824 |
| 9 | (CCTA or CCTAs or CTA or CTAs or CTCA or CTCAs or (CC adj2 TA) or ((CT or CTs or MDCT* or MSCT* or comput* tomograph*) adj CA)).tw,kf. | 12111 |
| **10** | **or/6-9 [CTA]** | **44279** |
| **11** | **5 and 10 [TAVI + CTA]** | **294** |
| **12** | **remove duplicates from 11** | **248** |

Database(s): Ovid MEDLINE(R) Epub Ahead of Print, In-Process & Other Non-Indexed Citations, Ovid MEDLINE(R) Daily and Ovid MEDLINE(R) 1946 to Present. Search performed on 23-12-2017
