# Supplementary material for: CTCA for detection of significant coronary artery disease in routine TAVI work-up: A systematic review and meta-analysis
Source: Neth Heart J. 2018 Sep 3;26(12):591–9. doi: 10.1007/s12471-018-1149-6 (PMC6288031; doi:10.1007/s12471-018-1149-6)
Supplement: Supplementary file 2 — Suppl. Table 2 Search (EMBASE) [file 12471_2018_1149_MOESM2_ESM.doc]

**Supplementary Table 2 Search (EMBASE)**

| **#** | **Searches** | **Results** |
| --- | --- | --- |
| 1 | transcatheter aortic valve implantation/ | 14400 |
| 2 | (TAVI or T-AVI or preTAVI or TA-ViVI or TAViVI or TA-AVI or TF-AVI or TFAVI or TAP-API or TAVR or TAVRs or preTAVR or TA-AVR or TFAVR or TF-AVR or TV-AVI or TVAVI or TAOAVI* or TAO-AVI* or TV-AVR* or TVAVR* or PAVI or P-AVI or PAVR or P-AVR).tw,kw. | 11418 |
| 3 | (TATM or T-AVR or THV).tw,kw. and (aort* or (heart valve adj2 (implant* or replac* or preimplant*))).mp. | 439 |
| 4 | ((percutan* or per-cutan* or transcutan* or trans-cutan* or transcath* or trans-cath* or transapic* or trans-apic* or transfemor* or trans-femor* or transsubclav* or subclav* or transaort* or trans-aort* or transvasc* or transvasc*) adj4 (AVR or AVI or AVRs or AVIs or (aort* adj (valve or valves) adj4 (implant* or preimplant* or replac* or intervent* or insert* or repair*)))).tw,kw,hw. | 16447 |
| 5 | or/1-4 [TAVI I] | 17063 |
| 6 | computed tomographic angiography/ | 42736 |
| 7 | (*computer assisted tomography/ or multidetector computed tomography/ or spiral computer assisted tomography/ or *computed tomography scanner/ or ((computed adj3 tomogra*) or variable helical pitch or (VHP adj3 (imag* or scan*)) or ((dual-source or high pitch or highpitch or multimodalit* or multidetector* or multislic* or (multi* adj3 (slic* or detector*))) adj6 (tomogra* or CT or CTs)) or MDCT* or MSCT*).tw,kw.) and (angiocardiography/ or coronary angiography/ or ((coronar* or non-invasiv* or noninasiv*) adj3 angiogra*).tw,kw.) | 14692 |
| 8 | (((comput* adj2 tomograph*) or CT or CTs or MDCT* or MSCT*) adj3 angio*).tw,kw,hw. | 65330 |
| 9 | (CCTA or CCTAs or CTA or CTAs or CTCA or CTCAs or (CC adj2 TA) or ((CT or CTs or MDCT* or MSCT* or comput* tomograph*) adj CA)).tw,kw. | 20129 |
| 10 | or/6-9 [CTA] | 77768 |
| 11 | 5 and 10 [TAVI/TAVR + CTA] | 765 |
| 12 | remove duplicates from 11 | 709 |
| 13 | 12 not medline.cr. | 698 |

Database(s): Embase Classic + Embase 1947 to 2017 December 22. Search performed on 23-12-2017
