# Supplementary material for: CTCA for detection of significant coronary artery disease in routine TAVI work-up: A systematic review and meta-analysis
Source: Neth Heart J. 2018 Sep 3;26(12):591–9. doi: 10.1007/s12471-018-1149-6 (PMC6288031; doi:10.1007/s12471-018-1149-6)
Supplement: Supplementary file 3 — Suppl. Table 3 CT scan characteristics (Supplement) [file 12471_2018_1149_MOESM3_ESM.doc]

**Supplementary Table 3 CT scan characteristics (Supplement)**

|  | **Contrast protocol** | **Spec. Contrast protocol (ml)** | **Flow rate**  **(ml/s)** | **Preheating of contrast** | **Slice thickness**  **(mm)** | **Collimation**  **(mm)** | **pitch** | **kernel** |
| --- | --- | --- | --- | --- | --- | --- | --- | --- |
| Pontone, 2011 | Triphasic injection | 80 contrast  50 saline  50 contrast | 5  5  3.5 | NR | 0.625 | NR | NR | NR |
| Andreini, 2014 | Triphasic injection | 80 contrast  50 saline  50 contrast | 5  5  3.5 | NR | 0.625 | NR | NR | NR |
| Hamdan, 2014 | Biphasic injection | 65-80 contrast  30 saline | 3.5  5 | NR | 0.625 | 96 x 0.625 | 0.2 | NR |
| Opolski, 2014 | Biphasic injection | 80-120 contrast  50 saline | 4  NR | NR | 0.6 | NR | NR | kernel |
| Harris, 2015 | Triphasic protocol | 40 contrast  30 contrast 2:1 saline  40 saline | 5  3  5 | NR | NR | NR | NR | I26f  I46f |
| Matsumoto, 2016 | Biphasic injection | *  20 saline | **  ** | NR | 0.5 | 100 x 0.5 | 0.15–0.17 | NR |
| Rossi, 2017 | Triphasic protocol | 50 contrast  30 contrast  50 saline | 5  3.5  3.5 | NR | 0.6 | 2 x 64 x 0.6 | NR | B26f |

mm = millimetre, ml = millilitre. Matsumoto described an algorithm for contrast volume administration: scan time x patient weight x 0.06 (*), and flow rate: patient weight x 0.06 mL/s (**).
