# Supplementary material for: CTCA for detection of significant coronary artery disease in routine TAVI work-up: A systematic review and meta-analysis
Source: Neth Heart J. 2018 Sep 3;26(12):591–9. doi: 10.1007/s12471-018-1149-6 (PMC6288031; doi:10.1007/s12471-018-1149-6)
Supplement: Supplementary file 4 — Suppl. Table 4 Methodological quality assessment of included studies by QUADAS-2 [file 12471_2018_1149_MOESM4_ESM.doc]

# Supplementary Table 4 Methodological quality assessment of included studies by QUADAS-2

|  | **Risk of Bias** | | | | **Applicability concerns** | | |
| --- | --- | --- | --- | --- | --- | --- | --- |
|  | **Patient-selection** | **Index Test** | **Reference standard** | **Flow and timing** | **Patient-selection** | **Index Test** | **Reference standard** |
| Pontone, 2011 | ☹ | ☺ | ? | ? | ☺ | ☺ | ☺ |
| Andreini, 2014 | ☹ | ☺ | ☺ | ☺ | ☺ | ☺ | ☺ |
| Hamdan, 2014 | ☹ | ☺ | ☺ | ☹ | ☺ | ☺ | ☺ |
| Opolski, 2014 | ☹ | ☺ | ☺ | ☺ | ☺ | ☺ | ☺ |
| Harris, 2015 | ? | ☺ | ? | ☺ | ☺ | ☺ | ☺ |
| Matsumoto, 2016 | ? | ☺ | ? | ☺ | ☺ | ☺ | ☺ |
| Rossi, 2017 | ☹ | ☺ | ☺ | ☹ | ☹ | ☺ | ☺ |

**Symbols represents the reported risk of bias or the concerns about applicability**. ☺ = low risk, ☹ = high risk, ? = unclear risk. QUADAS-2 = Quality Assessment of Studies of Diagnostic Accuracy Included in Systematic Reviews 2
