# Supplementary material for: CTCA for detection of significant coronary artery disease in routine TAVI work-up: A systematic review and meta-analysis
Source: Neth Heart J. 2018 Sep 3;26(12):591–9. doi: 10.1007/s12471-018-1149-6 (PMC6288031; doi:10.1007/s12471-018-1149-6)
Supplement: Supplementary file 5 — Suppl. Table 5 Summary of inclusion and exclusion of patients [file 12471_2018_1149_MOESM5_ESM.doc]

**Supplementary Table 5 Summary of inclusion**  and exclusion of patients

|  | **Patients referred for TAVI** | **CT+CAG**  **in TAVI work-up** | **Patients included in study** | **Patients**  **excluded**  **(%)** | **Reasons** |
| --- | --- | --- | --- | --- | --- |
| Pontone, 2011 | 80 | 80 | 60 | 20  *25%* | Atrial fibrillation, n = 10  Cardiac arrhythmias, n = 2  Inability to maintain breath hold, n = 1  Impaired renal function, n = 2  Heart rate > 70/min despite Ivabradine, n = 5 |
| Andreini, 2014 | 363 | 363 | 325 | 38  *10,5%* | Hypersensitivity to contrast agents, n = 3  Impaired renal function, n = 6  Inability to maintain breath hold, n = 8  Cardiac arrhythmias, n = 16  Heart rate > 70/min despite Ivabradine, n = 5 |
| Hamdan, 2014 | 135 | 135 | 115 | 20  *14,8%* | Long delay (>1 year) between CT and CAG, n = 10  Left ventricular assist device, n = 2  Inability to maintain breath hold, n = 8 |
| Opolski, 2014 | 645 | 510 | 475 | 170  *26,4%* | No CT and CAG performed, n = 135  Significant motion artefacts, n = 21  Poor contrast opacification, n = 14 |
| Harris, 2015 | ? | 100 | 100 | 0  *?* | No data available about selection pre-CT |
| Matsumoto, 2016 | ? | 90 | 60 | 30  *?* | No data available about selection pre-CT  No VHP scan protocol**, n = 30 |
| Rossi, 2017 | 339 | 250 | 140 | 199  *58,7%* | No CT and CAG performed, n = 89  Previous coronary revascularization*, n = 110 |
| **Total** | **1752** | **1528** | **1275** | **477**  ***27,2%*** |  |

The table lists all patients referred for transcatheter aortic valve replacement (TAVI), who underwent both computed tomography (CT) and coronary angiography (CAG) in the TAVI work-up, who were included in the study and who were excluded from the study. The excluded patients are shown as a number and as a percentage of the patients referred for TAVI. Furthermore, reasons for exclusion are listed in the last column. * = One study excluded all patients with known coronary artery disease (CAD), ** One study evaluated variable helical pitch (VHP) protocol and excluded patients without VHP protocol.
