# Supplementary material for: CTCA for detection of significant coronary artery disease in routine TAVI work-up: A systematic review and meta-analysis
Source: Neth Heart J. 2018 Sep 3;26(12):591–9. doi: 10.1007/s12471-018-1149-6 (PMC6288031; doi:10.1007/s12471-018-1149-6)
Supplement: Supplementary file 6 — Supplementary text—Risk of bias within studies [file 12471_2018_1149_MOESM6_ESM.docx]

**Supplementary text - Risk of bias within studies**

Overall, the selected studies showed excellent quality in terms of applicability. Risk of bias within the studies was scored as acceptable quality. Quality assessment of individual studies is visible in supplementary table 4 and is summarised in Fig. 2. All studies defined CTCA as the index test and blinded CTCA reviewers for coronary angiography outcome.(10–16) The subject of index test was consequentially rated as low risk of bias. Insufficient data was made available about patient selection in two studies (10,12) and scored unclear risk of bias for patient selection. In and exclusion criteria could have resulted in a selected patient population in 5 studies and scored a high risk of bias for patient selection.(11,13–16) Three studies did not explicitly mention blinding the performer of coronary angiography to the outcome of CTCA and scored unclear on the topic of reference standard.(10,12,14) Two studies were regarded as high risk of bias for flow and timing. These two studies excluded patients with known CAD (11) or maintained an interval of 1 year between index and reference test.(15) One study was unclear about flow and timing.(14) Regarding applicability, there were low concerns about applicability regarding the index and the reference test. Exclusion of patients with known CAD resulted in a high concern regarding applicability on the subject of patient selection.(11) All other papers scored low concern, regarding applicability on the subject of patient selection. The time between CTCA and coronary angiography differed between studies and is summarised schematically in Fig. 3. All inclusion and exclusion criteria of the individual studies and the studies combined are listed in supplementary Table 5 of the supplementary material and summarised in supplementary Fig. 1 of the supplementary material.

**Figure Legends supplementary material**

**Supplementary Fig. 1 Total inclusion and exclusion of patients**

Fig. 1 shows the difference in patients referred for TAVI and patients included in final analysis. Reasons for exclusion are listed with corresponding amount of patients.

* One study excluded all patients with known coronary artery disease.

** One study evaluated VHP protocol and excluded patients without VHP protocol.

*CAD* coronary artery disease, *CTCA* computed tomography coronary angiography, *TAVI* transcatheter aortic valve implantation, *VHP* variable helical pitch

**Supplementary Fig. 2 Subgroup analysis for CT-scanner rotation times**

Fig. 2 shows the subgroup analysis for the CT-scanner rotation times. Sensitivity versus 1‐specificity is plotted in two summary receiver operator characteristic curves for studies reporting on CT scanners with >300 ms rotation time (Golden line, golden square) and <300 ms rotation time (Red line, red square). Each study is represented by a coloured circle, size being dependent on study size. The coloured square represents the summary estimate, the colour corresponds with the appurtenant line and square. The thick dashed lines represents the 95% confidence region.

*Sym* symbol

**Supplementary Fig. 3 Subgroup analysis for CAD prevalence**

Fig. 3 shows the subgroup analysis for the prevalence of CAD. Sensitivity versus false positive rate is plotted in two summary receiver operator characteristic curves (SROC) for studies reporting on CAD prevalence of ≥50% (Green line, green square) and <50% (Blue line, blue square). Each study is represented by a coloured circle, size being dependent on study size. The coloured square represents the summary estimate, the colour corresponds with the appurtenant line and square. The thick dashed lines represents the 95% confidence region. *Sym* symbol
