# Supplementary material for: CTCA for detection of significant coronary artery disease in routine TAVI work-up: A systematic review and meta-analysis
Source: Neth Heart J. 2018 Sep 3;26(12):591–9. doi: 10.1007/s12471-018-1149-6 (PMC6288031; doi:10.1007/s12471-018-1149-6)
Supplement: Supplementary file 7 — Suppl. Fig. 1 Total inclusion and exclusion of patients [file 12471_2018_1149_MOESM7_ESM.doc]

**Referral**

**Screening**

**Included**

**Patients in final analysis**

**1275**

**Patients referred for TAVI**

**1752**

**Patients excluded, n = 477**

No CT and CAG performed (n=224)

Previous coronary revascularization* (n=110)

No VHP scan protocol** (n=30)

Significant motion artefacts n=21)

Cardiac arrhythmias (n=18)

Inability to maintain breath hold (n=17)

Poor contrast opacification (n=14)

Atrial fibrillation (n=10)

Long delay (>1 year) between CT and CAG (n=10)

Heart rate > 70/min despite Ivabradine (n=10)

Impaired renal function (n=8)

Hypersensitivity to contrast agents (n=3)

Left ventricular assist device (n=2)
