# Supplementary figures and images for: CTCA for detection of significant coronary artery disease in routine TAVI work-up: A systematic review and meta-analysis
Source: Neth Heart J. 2018 Sep 3;26(12):591–9. doi: 10.1007/s12471-018-1149-6 (PMC6288031; doi:10.1007/s12471-018-1149-6)

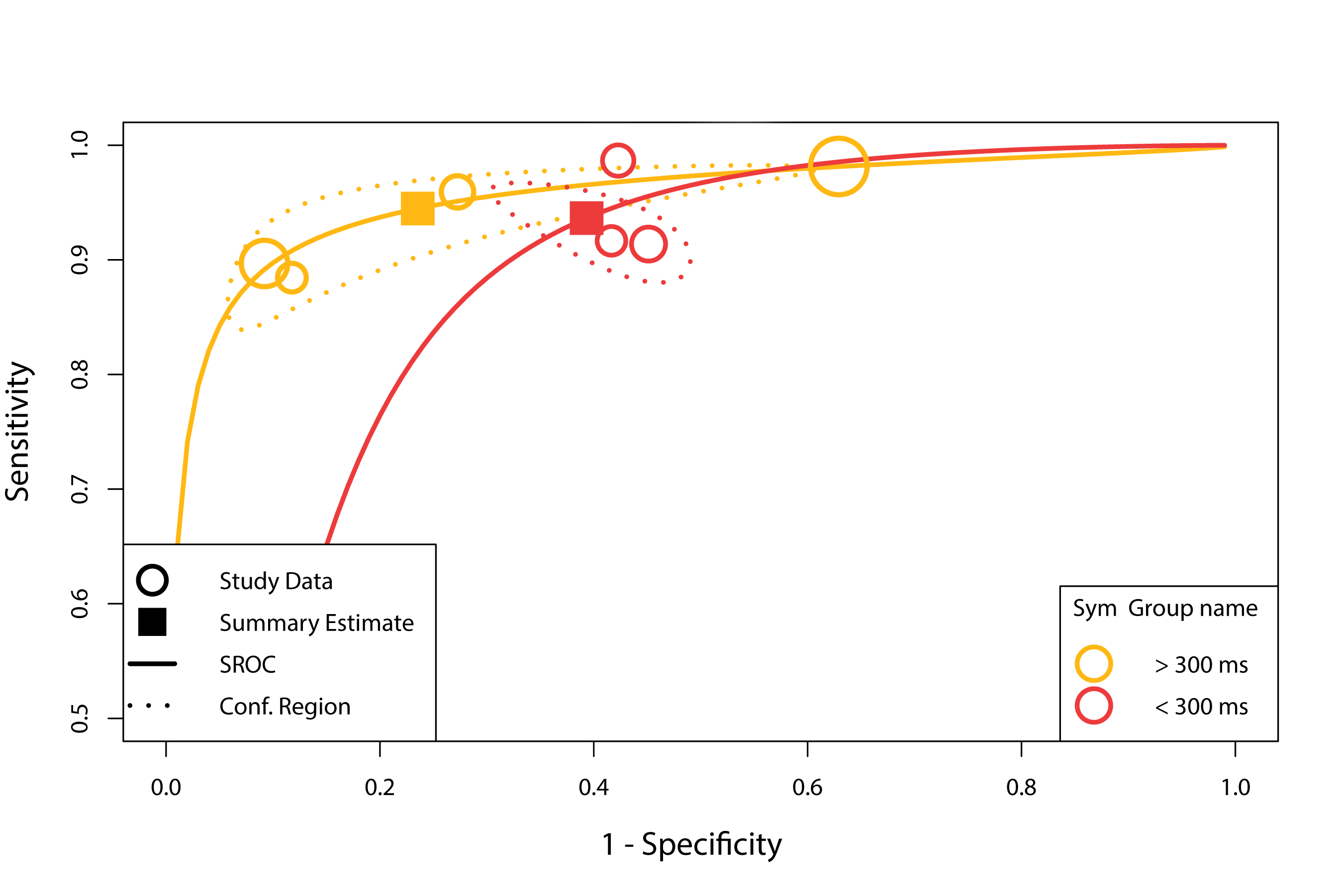

Supplement: Supplementary file 8 — Suppl. Fig. 2 Subgroup analysis for CT-scanner rotation times [file 12471_2018_1149_MOESM8_ESM.tif]

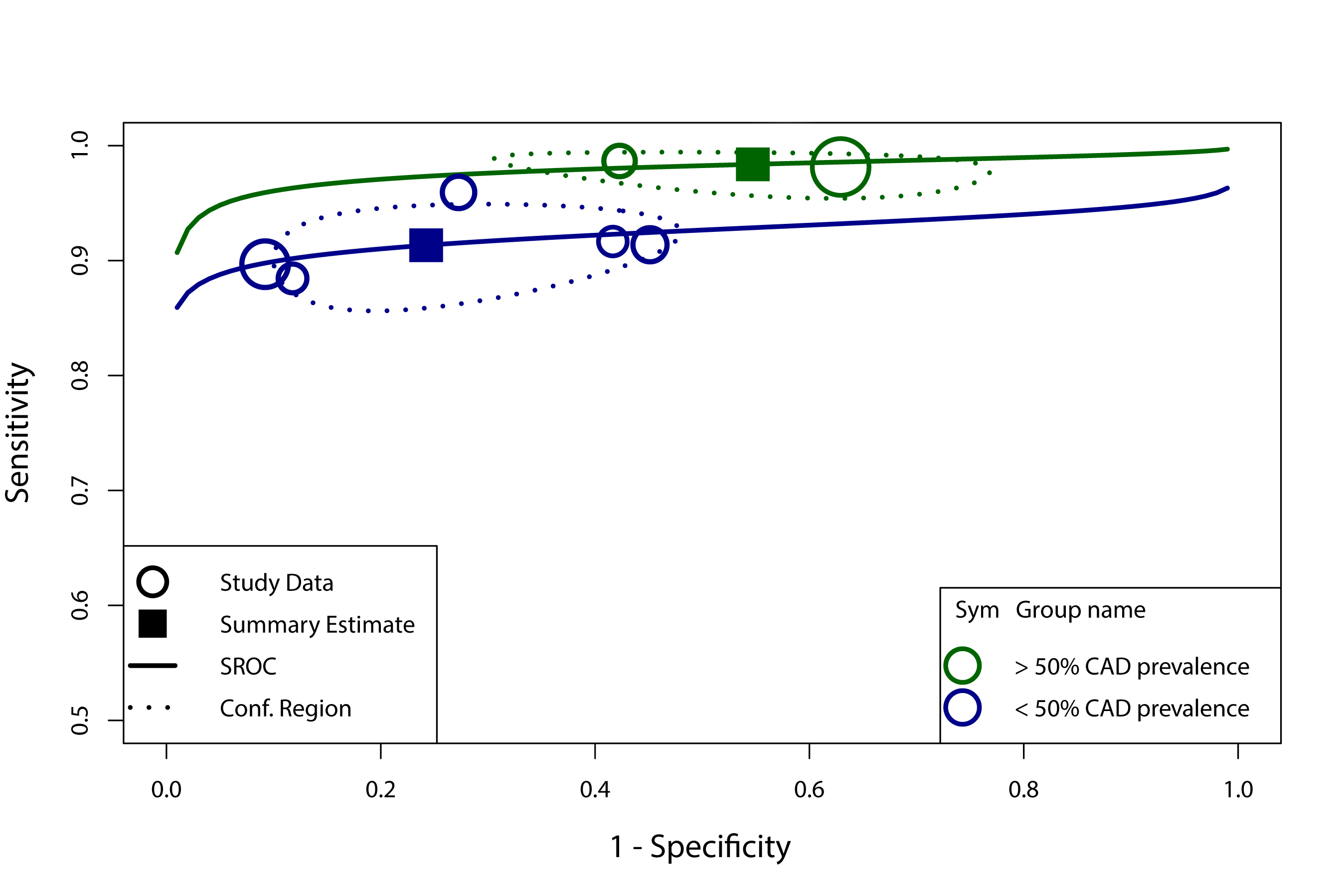

Supplement: Supplementary file 9 — Suppl. Fig. 3 Subgroup analysis for CAD prevalence [file 12471_2018_1149_MOESM9_ESM.tif]
